# Synthetic applications of gold-catalyzed ring expansions

**DOI:** 10.3762/bjoc.7.87

**Published:** 2011-06-07

**Authors:** David Garayalde, Cristina Nevado

**Affiliations:** 1 Organic Chemistry Institute, University of Zürich, Winterthurerstr. 190, CH-8057, Zürich, Switzerland

**Keywords:** cycloisomerizations, gold, homogeneous catalysis, ring expansion, strained rings

## Abstract

The development of new methodologies catalyzed by late transition metals involving cycloisomerizations of strained rings can open new venues for the synthesis of structurally complex molecules with interesting biological activities. Herein we summarize, from both a synthetic as well as a mechanistic point of view, the most recent developments in gold-catalyzed ring expansions.

## Introduction

Over the past twenty years, the image of gold has evolved, from being considered a dead-entity in terms of chemical reactivity, to playing a key role in catalytic processes. The vast array of gold-mediated transformations reported so far share a common feature: The ability of gold(I) and gold(III) species to activate unsaturated moieties due to the strong relativistic effects governing its coordination behavior [1–6]. However, beyond its Lewis acidity properties towards alkynes, allenes or alkenes, gold has also proved to be extremely powerful in triggering ring-expansion processes to introduce structural complexity into organic molecules. The gold-catalyzed ring expansion of strained rings is viewed nowadays as a flexible synthetic tool in organic synthesis [7–9].

In this review, we aim to summarize the most recent developments in gold-catalyzed ring expansions, from both a synthetic and a mechanistic point of view. A deeper understanding of the processes governing gold-chemistry allows organic chemists to become more creative in designing novel processes, which might provide access to architectures that were so far inaccessible.

**Scheme 1:**
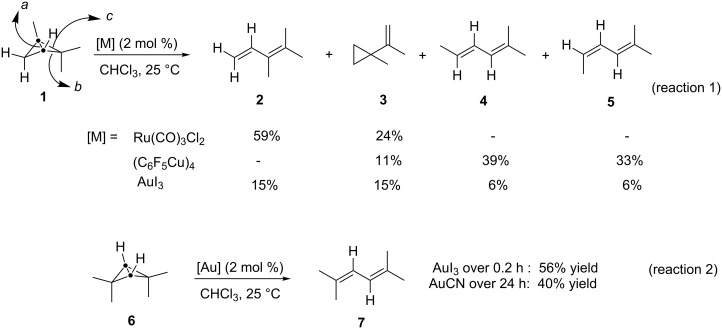
Transition metal promoted rearrangements of bicyclo[1.1.0]butanes.

After the first examples on the perchlorination of naphthalene with AuCl_3_ or AuCl by Schwemberger and Gordon in 1935 [10], almost forty years passed without a single report on the catalytic ability of gold salts, due to its presumed lack of reactivity. In 1972, Paul G. Gassman reported several studies on transition metal promoted rearrangements of bicyclo[1.1.0]butanes [11]. Thus Ru–carbonyl complexes promote the rearrangement of 1,2,2-trimethylbicyclo[1.1.0]butane (**1**) to yield diene **2** and the cyclopropyl derivative **3** (Scheme 1, reaction 1: a,b for **2** and **3**), whilst pentafluorophenylcopper tetramer affords predominately dienes **4** and **5** (Scheme 1, reaction 1: c for **4** and **5**). By contrast, gold salts show almost no preference for the activated C–C sigma bond in the substrate. However, in the case of 2,2,4,4-tetramethylbicyclo[1.1.0]butane (**6**), the reaction was completely selective and gave 2,5-dimethyl-2,4-hexadiene (**7**) in moderate yields when either AuI_3_ or AuCN were used as catalysts (Scheme 1, reaction 2).

**Scheme 2:**
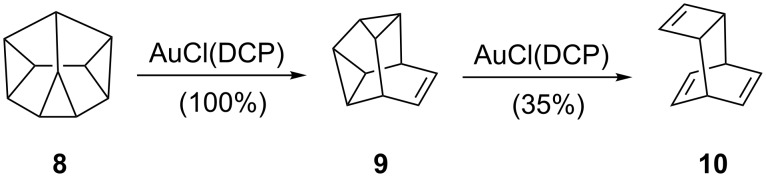
Gold-catalyzed rearrangements of strained rings.

Only four years later, de Meijere reported a gold-catalyzed rearrangement of strained small ring hydrocarbons [12]. Although heterogeneous catalysis seemed to be operating in this case, homogeneous complexes such as AuCl(DCP) (DCP = dicyclopentadiene) were able to trigger the quantitative rearrangement of diademane (**8**) to snoutene (**9**) and, at least partially, the rearrangement of the latter into basketene (**10**) (Scheme 2).

Although the structures of the final products in these transformations are rather simple and the low selectivities limit the synthetic potential of these methods, the fact that gold was able to activate strained ring systems opened up a new research area that is still highly active to date, as will be shown in the following sections of this review.

## Review

### 1 Ring expansions involving oxygenated functions

#### 1.1 Cyclopropanols and cyclobutanols

**Scheme 3:**
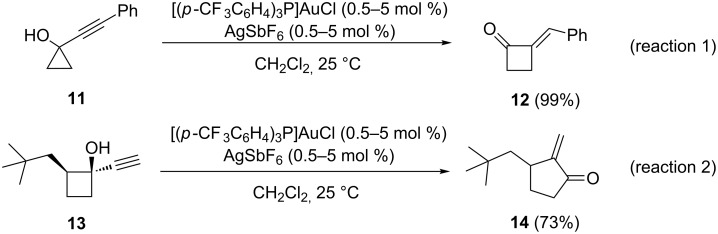
Gold-catalyzed ring expansions of cyclopropanols and cyclobutanols.

Substituted cyclobutanones [13–15] and cyclopentanones [16–18] constitute valuable building blocks in organic synthesis due to their rich chemistry. In addition, they are common motifs in numerous natural products. Among the various approaches to access these ubiquitous scaffolds, the gold(I)-catalyzed ring expansion of cyclopropanols and cyclobutanols is considered one of the most powerful and versatile methods. In 2005, Toste and co-workers reported the treatment of 1-(phenylethynyl)cyclopropanol (**11**) with tris(4-trifluoromethylphenyl)phosphine gold(I) to give alkylidenecyclobutanone **12** quantitatively (Scheme 3, reaction 1) [19]. In an analogous manner, alkynylcyclobutanols were suitable substrates for gold(I)-catalyzed ring expansions only when a terminal alkyne group was present (Scheme 3, reaction 2). Thus, cyclobutanol **13** gave 2-methylene-3-neopentylcyclopentanone (**14**) in 73% yield.

**Scheme 4:**
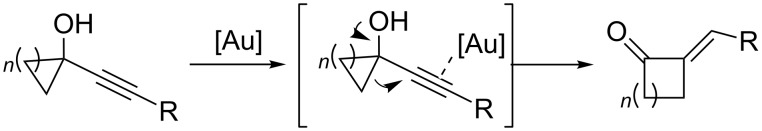
Mechanism of the cycloisomerization of alkynyl cyclopropanols and cyclobutanols.

Both processes were rationalized as a result of the π-activation of the alkyne in the presence of gold, followed by migration of the C–C bond, and a final 1,4-H shift (Scheme 4) [20].

**Scheme 5:**
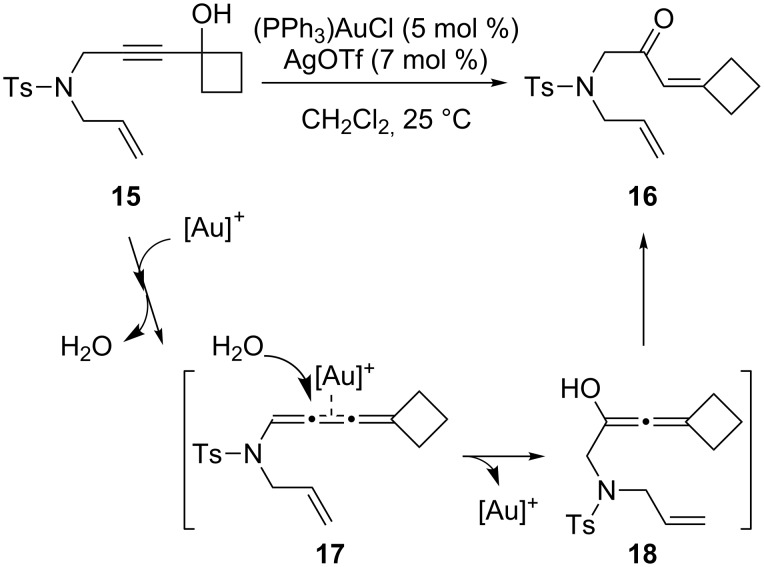
Proposed mechanism for the Au-catalyzed isomerization of alkynyl cyclobutanols.

Interestingly, the use of internal alkynyl cyclobutanols such as **15**, reported in 2007 by Chung and co-workers [21], led to a completely different outcome (Scheme 5). This transformation did not lead to the expected cyclopentanones. Instead, α,β-unsaturated ketones **16** were isolated in good yields. The proposed mechanism (Scheme 5) involves nucleophilic attack by a molecule of water on the activated alkyne moiety, followed by dehydration to give the cumulene intermediate **17**. Attack on **17** by a second water molecule regenerates the catalyst with the formation of intermediate **18**, which then tautomerizes to afford the observed product.

**Scheme 6:**
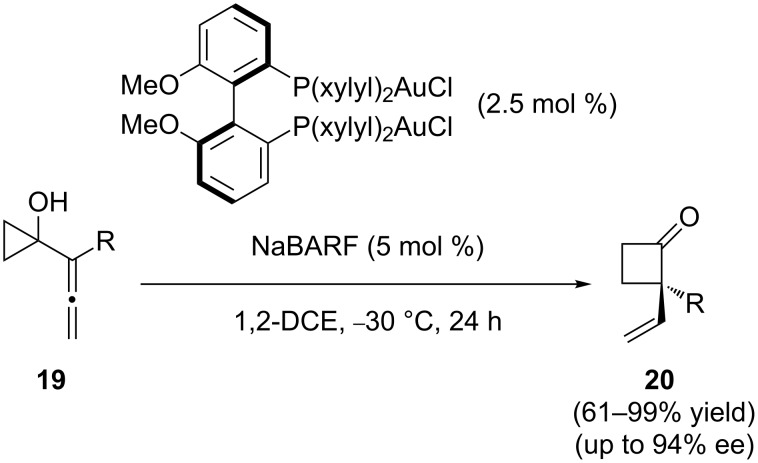
Gold-catalyzed cycloisomerization of 1-allenylcyclopropanols.

1-Allenyl cyclopropanols **19** can be transformed into cyclobutanones **20** with absolute stereocontrol at the quaternary stereogenic center generated during the reaction by the use of a binuclear chiral gold-phosphine complex, as shown in Scheme 6 [22]. Bicyclic cyclopentanones can also be obtained in a related transformation starting from allenyl cyclobutanols [23].

#### 1.2 Cyclopropylmethanols

**Scheme 7:**
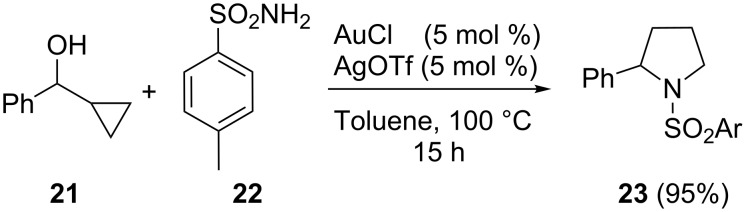
Gold-catalyzed cycloisomerization of cyclopropylmethanols.

Cyclopropyl methanols can be used, alternatively, as pre-electrophiles in gold-catalyzed reactions. In 2008 Chan and co-workers developed an efficient synthetic route to pyrrolidines via a tandem amination/ring expansion of these substrates in the presence of sulfonamides [24]. Phenylcyclopropylalcohol **21** was efficiently transformed into sulfonyl pyrrolidine **23** in the presence of 5 mol % of the cationic complex AuOTf (Scheme 7). The reaction was applicable to a wide range of activated and non-activated cyclopropylmethanols, sulfonamides containing electron-withdrawing, electron-donating, and sterically demanding substituents. This transformation is thought to proceed through activation of the substituted cyclopropylmethanol by the gold catalyst, which leads to the ionization of the alcohol followed by the subsequent cyclopropyl ring opening and trapping of the carbocation by the sulfonamide. Subsequent intramolecular hydroamination gave the pyrrolidine products.

#### 1.3 Oxiranes

**Scheme 8:**
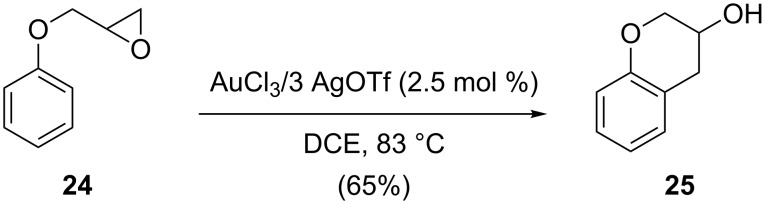
Gold-catalyzed cycloisomerization of aryl alkyl epoxides.

As an oxophilic Lewis acid, gold can activate epoxides towards the attack of nucleophiles. A good example is the AuCl_3_ catalyzed ring opening of aryl alkyl epoxide **24** to give 3-chromanol **25**, which was reported by He and co-workers in 2004 (Scheme 8) [25].

**Scheme 9:**
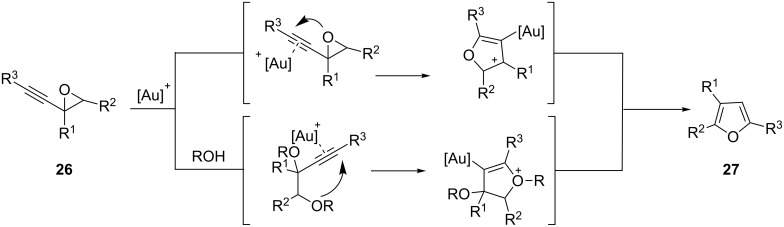
Gold-catalyzed synthesis of furans.

The same year, Hashmi and co-workers described the first example of a gold-catalyzed conversion of alkynyl epoxides **26** into furans **27** [26–27]. Mechanistic studies performed later by Pale and co-workers [28] seem to rule out the usually proposed mechanism, that is, via the intramolecular nucleophilic addition of the oxirane oxygen on the π-metal–alkyne complex (Scheme 9, upper row). Instead, the reaction seems to proceed through a cascade initiated by an internal or external nucleophilic (the hydroxy group in the substrate or adventitious water or alcohol present in the reaction media) opening of the three membered ring, followed by metal activation of the triple bond to trigger the cyclization (Scheme 9, lower row). In both cases, aromatization and protodeauration would afford the observed products.

**Scheme 10:**
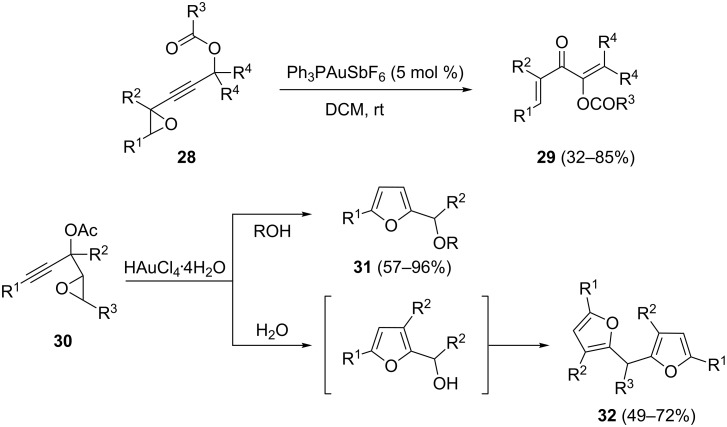
Transformations of alkynyl oxiranes.

Acyloxylated alkynyl oxiranes **28** and **30** have also proved to be versatile building blocks for the synthesis of divinyl ketones **29** [29], 2,5-disubstituted furans **31** [30] and difurylmethane derivatives **32** [31], respectively (Scheme 10).

**Scheme 11:**
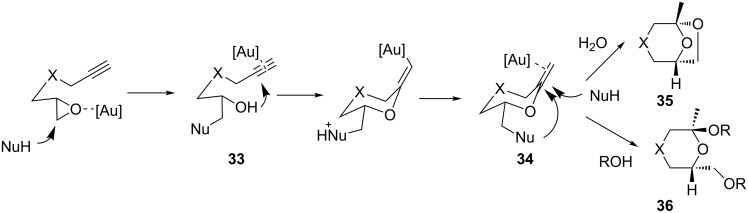
Transformations of alkynyl oxiranes into ketals.

Epoxy alkynes can also be transformed with high stereoselectivity into ketals in the presence of catalytic amounts of gold and an external nucleophile such as water or an alcohol (Scheme 11) [32]. The reaction seems to commence with the epoxide ring opening in the presence of the nucleophile to give intermediate **33** (as already proposed in Scheme 9) followed by activation of the alkyne and intramolecular nucleophilic attack of the alcohol function to give **34**. Reactivation of the olefin and subsequent incorporation of a second molecule of nucleophile (intramolecularly in the case of water, intermolecularly in the case of alcohols) affords ketals **35** and **36**, respectively. The reaction can also proceed in an intramolecular manner, if the substrate contains an alcohol functionality [33–34].

### 2 Ring expansions involving cyclopropyl alkynes

**Scheme 12:**
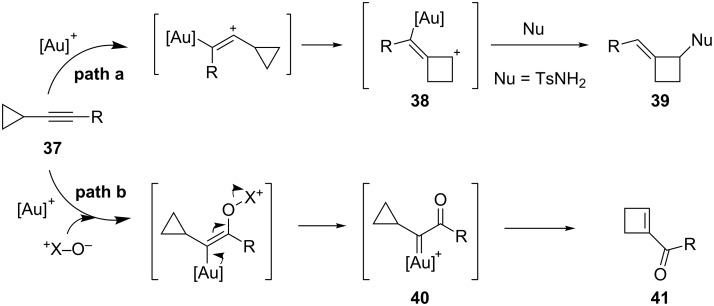
Gold-catalyzed cycloisomerization of cyclopropyl alkynes.

The metal-catalyzed ring expansion of cyclopropyl alkyne derivatives represents a versatile method to access a wide range of building blocks [35–38]. Upon gold activation of the triple bond in **37** two possible pathways can arise. In the first, the cyclobutyl cation **38** is formed by ring expansion, which is subsequently trapped by an external nucleophile (Scheme 12, path a). In 2010, the group of Yu developed a new route for the synthesis of cyclobutanamines **39** according to this reaction mode [39]. Alternatively, in the presence of an external oxidant, a nucleophilic addition can occur to form carbene **40**, which rearranges to cyclobutenone **41** (Scheme 12, path b). Liu recently reported the use of diphenylsulfoxide as an external nucleophilic oxidant in this context [40].

**Scheme 13:**
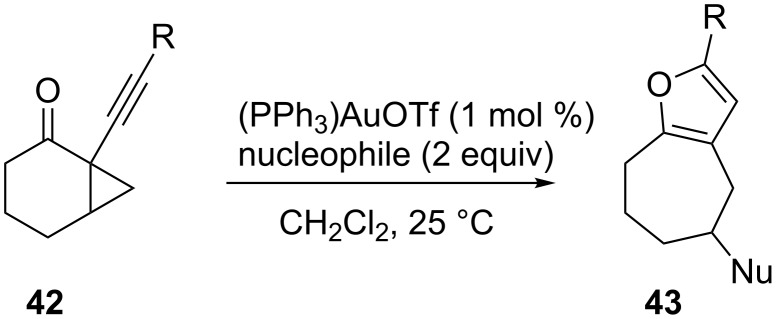
Gold-catalyzed synthesis of substituted furans.

The gold-catalyzed intramolecular nucleophilic attack of heteroatoms on alkynes, followed by ring expansion, represents an appropriate method for the synthesis of furans and pyrroles. In 2006, Schmalz and co-workers reported a gold-catalyzed cascade reaction of alkynyl cyclopropyl ketones **42**, which makes use of the carbonyl group as a nucleophile, and yields substituted furans **43** (Scheme 13) [41].

**Scheme 14:**
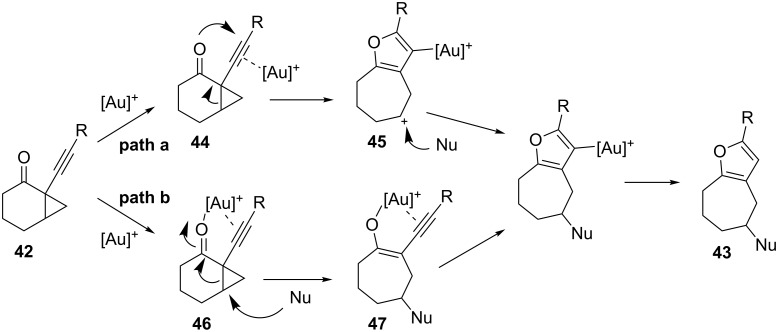
Proposed mechanism for the isomerization of alkynyl cyclopropyl ketones.

Two possible mechanisms were proposed for this transformation. Nucleophilic attack of the carbonyl oxygen atom onto the activated alkynyl moiety can trigger the cyclopropyl ring opening to give carbocation **45,** which is then trapped in the presence of an external nucleophile to give, after protonolysis, furans **43** (Scheme 14, path a). Alternatively, gold can complex both of the unsaturated moieties as in **46**, triggering the cyclopropyl ring opening through an intermolecular nucleophilic attack to give intermediate **47**, which upon cycloisomerization affords the aromatic product (Scheme 14, path b).

**Scheme 15:**
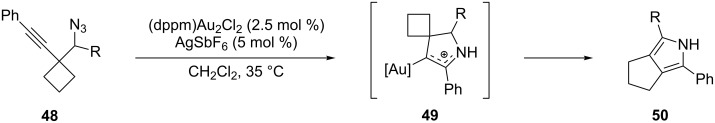
Cycloisomerization of cyclobutylazides.

Toste and co-workers reported an intramolecular acetylenic Schmidt reaction using azides as internal nucleophiles to give substituted pyrroles (Scheme 15) [42]. Gold activation of the alkyne in **48**, addition of the azide moiety followed by a loss of dinitrogen affords a gold-stabilized cationic intermediate **49**. A subsequent 1,2-H shift gave, after tautomerization, the 1*H*-pyrrole **50**. Epoxides can also be used as nucleophiles for the preparation of heterocarbocycles via gold-catalyzed ring expansion of 1-oxiranyl-1-alkynylcyclopropanes [43–44].

**Scheme 16:**
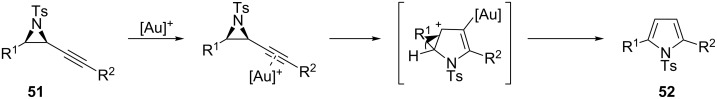
Cycloisomerization of alkynyl aziridines.

An alternative method for obtaining disubstituted pyrroles via gold-catalyzed ring expansion was reported by Davies and co-workers who employed alkynyl aziridines **51** as intramolecular nucleophiles [45]. Ring expansion from the aziridines onto the adjacent alkyne afforded the 2,5-disubstituted pyrroles **52** in high yields (Scheme 16).

**Scheme 17:**
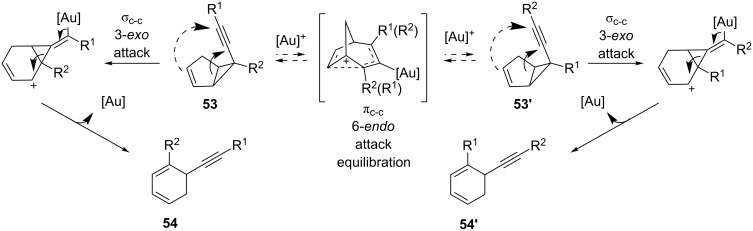
Gold-catalyzed synthesis of disubstituted cyclohexadienes.

In 2011, Barluenga et al. developed a new methodology for the preparation of 1,6-disubstituted regioisomeric cyclohexadienes **54** and **54'** (Scheme 17) [46]. The process resulted in a five-to-six-membered ring expansion which involves the cleavage of the bridging C–C bond and a formal [1,2]-alkynyl shift. A mixture of regioisomers resulted due to an unexpected equilibration of the starting material **53** to **53'** via 6-*endo* cyclization of the olefin with the gold-activated alkyne.

### 3 Ring expansions of cyclopropenes

**Scheme 18:**
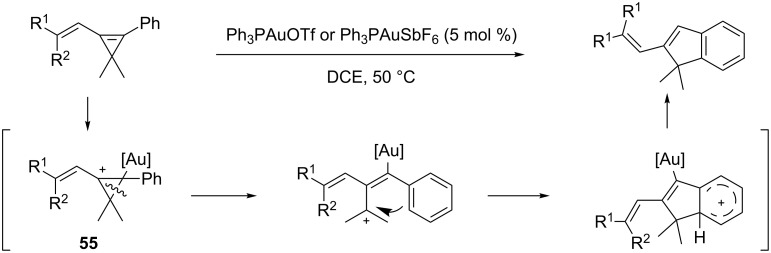
Gold-catalyzed synthesis of indenes.

Highly strained cyclopropenes can undergo a wide variety of transformations in the presence of Lewis acids. Shi and co-workers reported in 2008 a gold-catalyzed cycloisomerization of aryl vinyl cyclopropenes to produce, selectively, 2-vinyl-1*H*-indene derivatives in high yields (Scheme 18). Upon activation of the cyclopropene, cation **55** is formed. C–C bond cleavage of the cyclopropyl ring followed by a Friedel–Crafts reaction affords, after recovery of aromaticity, the observed products [47].

### 4 Ring expansions involving annulation reactions

**Scheme 19:**
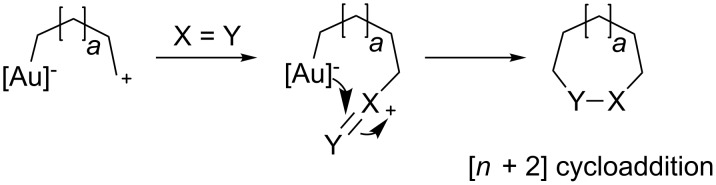
Gold-catalyzed [*n* + *m*] annulation processes.

Diels–Alder, [1,3]-dipolar-, [2 + 2]- and [4 + 3]-cycloadditions are just some of the relevant available methods employed by organic chemists to increase the molecular complexity of products originating from rather straightforward starting materials. In contrast to the vast number of precedents involving Rh-catalyzed [4 + 3]-cycloaddition reactions to form 7-membered-rings in a stereocontrolled manner [48–49], the use of the analogous gold-catalyzed transformations has remained, until recently, largely unexplored. Usually, 1,*n*-dipoles are elusive intermediate species, which can undergo many side reactions preceding the desired annulation/cyclization processes. Zhang envisioned that if the negative terminus of the dipole could be stabilized in the presence of gold, a better handling of these species could be achieved to trigger [*n* + *m*] annulation processes. In fact, the cationic end of the dipole was proposed to react in a bimolecular process in the presence of a dipolarophile, such that the nucleophilic C–Au bond could intercept the newly generated delta positive charge (Scheme 19).

**Scheme 20:**
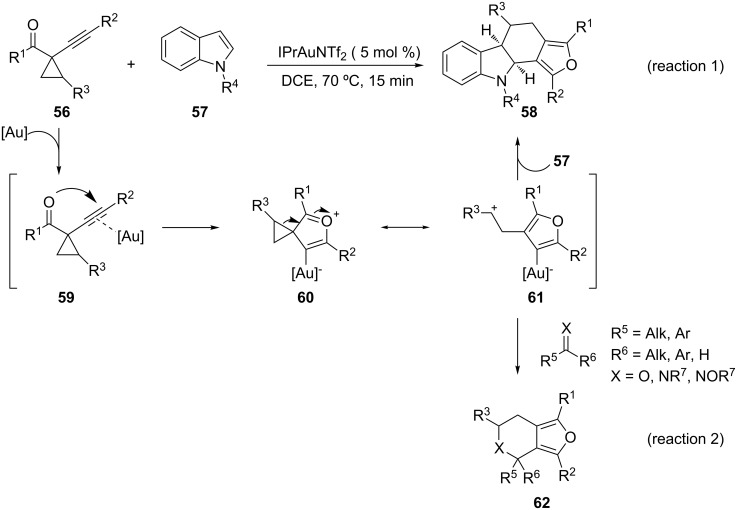
Gold-catalyzed generation of 1,4-dipoles.

This concept, successfully applied to self cyclization processes [50], could be enforced in its intermolecular version by generation of gold-1,4-dipoles, which minimize self cyclization events [51]. 1-(1-Alkynyl)cyclopropyl ketones **56** proved to be versatile building blocks for this purpose and gave, in the presence of indoles **57** as dipolarophiles, tetracyclic furans **58** in excellent yields (Scheme 20, reaction 1). NHC carbenes are preferred as ancillary ligands on the metal center. Upon coordination of the metal to the alkyne **59**, the 1,4-dipole **61** can be formed from oxocarbenium **60**. Carbonyl compounds and carbonyl derivatives, such as imines or silyl enol ethers, can also be used as dipolarophiles to generate bicyclic furans **62** in fairly good yields (Scheme 20, reaction 2). Nitrones also reacted as dipolarophiles in the presence of AuCl_3_, even if in some cases copper catalysts were found to be more effective at triggering the corresponding annulations [52].

**Scheme 21:**
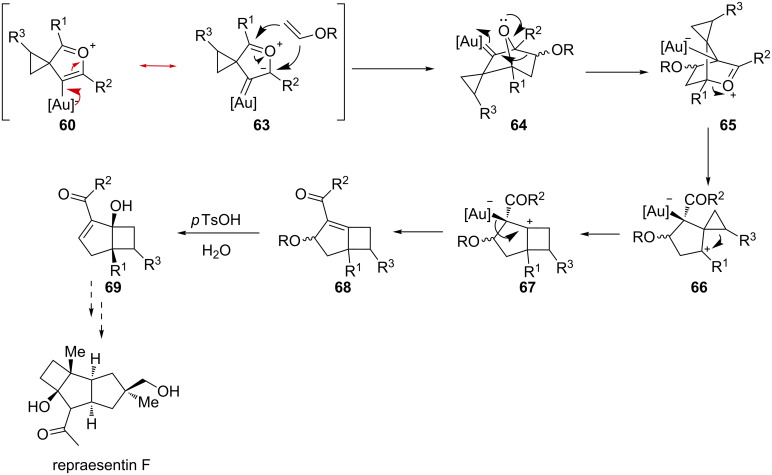
Gold-catalyzed synthesis of repraesentin F.

By contrast, when alkoxy vinyl ethers were employed as dipolarophiles, the cycloaddition takes place prior to the formation of the 1,4-furan dipole (Scheme 21). In fact, a resonance structure of **60** can be envisaged entailing a gold–carbene and a carbonyl ylide **63**. Upon 1,3-dipolar cycloaddition with the alkoxy vinyl ether, bridged bicycle **64** is formed. 1,2-Alkyl migration and bridge opening produces a spiro cation **66**, such that a consecutive cyclopropyl ring expansion affords the bicyclic [3.2.0]heptane skeleton **68** in excellent yield and selectivity [53]. Treatment of **68** with a protic acid in water should activate the enone system triggering the nucleophilic attack of water to give hydroxy ketones **69**. The synthetic utility of the method can be easily recognized by an examination of the structure of natural products such as repraesentin F, whose core largely comprises the structural motifs generated in this gold-catalyzed cascade.

### 5 Ring expansions involving enynes

**Scheme 22:**
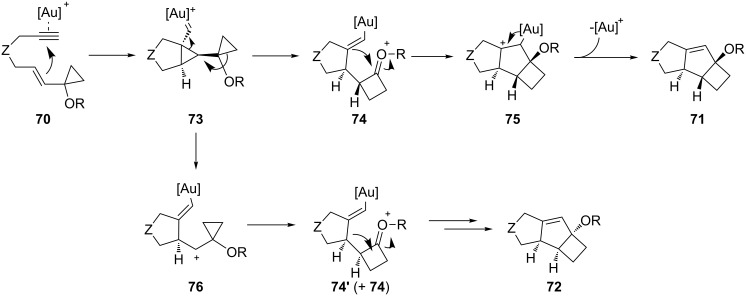
Gold-catalyzed ring expansion of cyclopropyl 1,6-enynes.

The gold-catalyzed heteroatom-assisted 1,2-shift already summarized in section 1 of this review, can offer further synthetic potential in combination with 1,6-enyne substrates. Echavarren successfully developed a gold-catalyzed Prins cyclization of enynes **70** to afford *trans*- and *cis*-octahydrocyclobuta[*a*]pentalene skeletons **71** and **72**, respectively (Scheme 22) [54]. In most cases *trans* products were favored. The reaction is proposed to proceed via the cyclopropyl carbene **73**, which undergoes ring expansion to form the alkenyl–gold intermediate **74**. Reaction of the latter with the oxonium cation produces **75**, which upon gold departure forms tricycles **71**. If a non-concerted process takes place, then cyclopropyl carbene **73** evolves towards cyclopropyl cation **76**, which upon non-stereospecific ring expansion and cyclization could explain the formation of both *cis* and *trans* reaction products **71** and **72**, respectively.

**Scheme 23:**
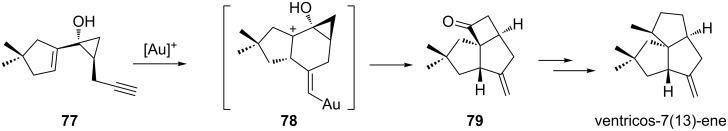
Gold-catalyzed synthesis of ventricos-7(13)-ene.

Toste and co-workers also reported a remarkable synthetic application of a gold-catalyzed ring expansion of cyclopropanols in enynic substrates [55]. Vinyl cyclopropanol **77** reacts with Ph_3_PAuBF_4_ via cyclization, followed by a selective semi-pinacol shift via carbocationic intermediate **78**, to give cyclobutanone **79**, which is readily transformed into the angular triquinane ventricosene in six steps (Scheme 23).

### 6 Ring expansions involving propargyl acyloxy rearrangements

**Scheme 24:**
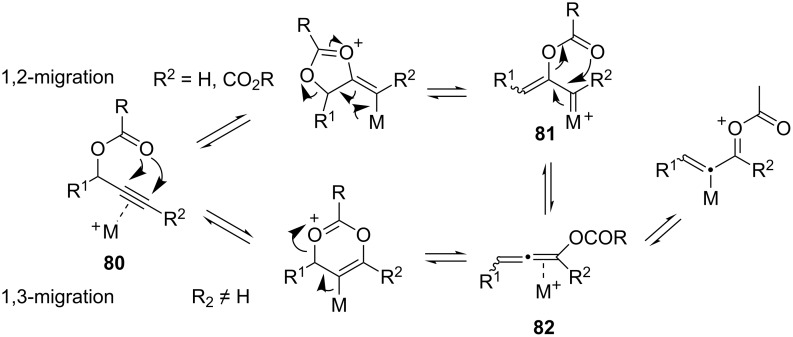
1,2- vs 1,3-Carboxylate migration.

Propargyl carboxylates **80** can be π-activated by gold towards 1,2-acyloxy migration and/or [3,3]-sigmatropic rearrangement. Two different, but mechanistically related, intermediates characterize these competitive processes, i.e., 1,2-migration via metal "carbenoid" **81** formation and [3,3]-sigmatropic rearrangement via allenyl acetate **82** as an intermediate (Scheme 24) [5,56–57].

**Scheme 25:**
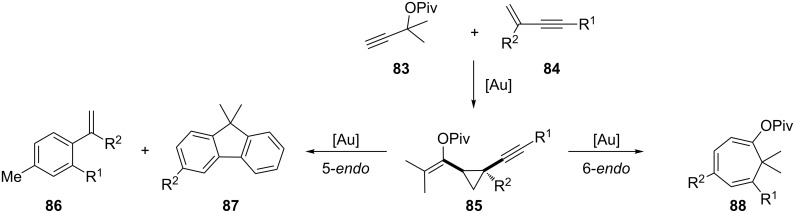
Gold-catalyzed cycloisomerization of vinyl alkynyl cyclopropanes.

In 2008, Toste and co-workers reported a gold(I)-catalyzed cycloisomerization of *cis*-pivaloyloxy vinyl alkynyl cyclopropanes **85** to give arenes **86**, **87** and cycloheptatriene **88** derivatives through 5-*endo-dig* and 6-*endo-dig* cyclization reactions, respectively, under careful control of the reaction conditions (Scheme 25) [58].

**Scheme 26:**
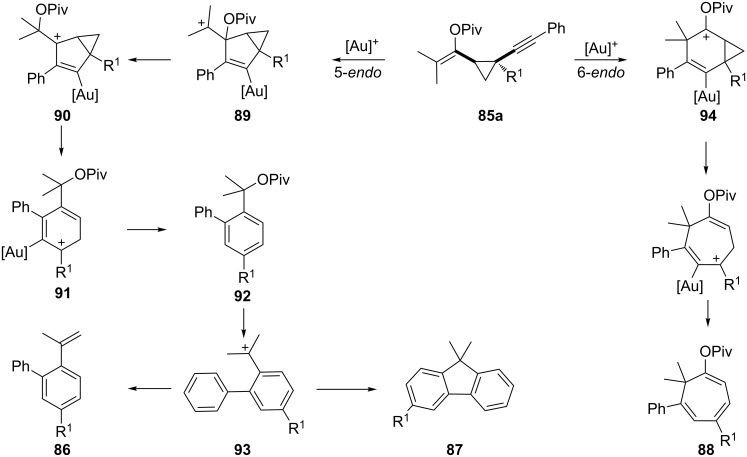
Proposed mechanism for the cycloisomerization of vinyl alkynyl cyclopropanes.

A mechanistic rationale for these transformations is shown in Scheme 26. Cyclopropanes **85a** are generated in situ by intermolecular cyclopropanation of enyne **84** and a carbene resulting from the rearrangement of propargyl ester **83**. When tertiary propargyl esters are used, the 5*-endo-dig* cyclization generates the carbocation **89**. Migration of the pivaloyloxy group affords the allylic cations **90** and **91** by delocalization of the positive charge onto gold. The aromatic intermediate **92** is probably converted, via **93**, into **86** and **87** by E1 and S_N_1 mechanisms, respectively. When secondary esters are employed, 6-*endo-dig* cyclization occurs to give **94**, which forms the cycloheptatriene derivate **88** upon cyclopropyl ring expansion.

**Scheme 27:**
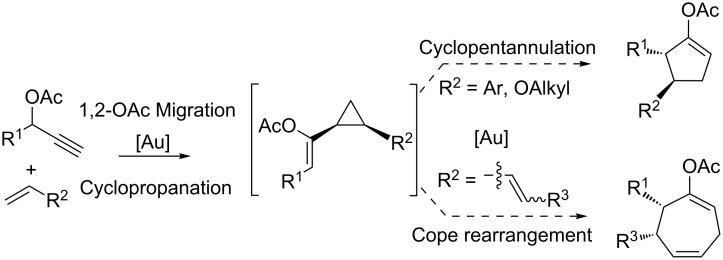
Gold-catalyzed 1,2-acyloxy rearrangement/cyclopropanation/cycloisomerization cascades.

**Scheme 28:**
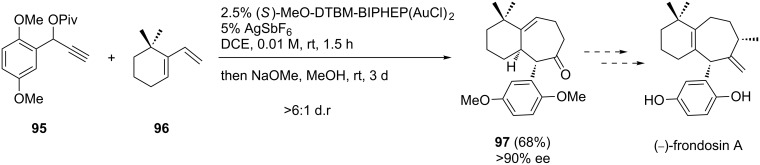
Formal total synthesis of frondosin A.

Nevado and co-workers [59] have also recently used cyclopropyl intermediates, generated in situ via alkene cyclopropanations mediated by gold carbenes, for the stereocontrolled synthesis of 5- and 7-membered-rings (Scheme 27). This method was subsequently applied in a formal enantioselective synthesis of frondosin A, a marine norsesquiterpenoid with promising biological activities (Scheme 28) [48]. Treatment of pivalate **95** and 6,6-dimethyl-1-vinylcyclohexene (**96**) with (*S*)-MeO-DTBM-BIPHEP-gold(I) complex afforded the corresponding bicyclic cycloheptenyl pivalate quantitatively. In situ hydrolysis and subsequent equilibration with NaOMe/MeOH yielded thermodynamically favored ketone **97** in 68% yield and >90% ee. Since this bicyclic enone has been recently elaborated to frondosins A and B [60–61] this approach represents a streamlined formal enantioselective synthesis of both molecules.

**Scheme 29:**
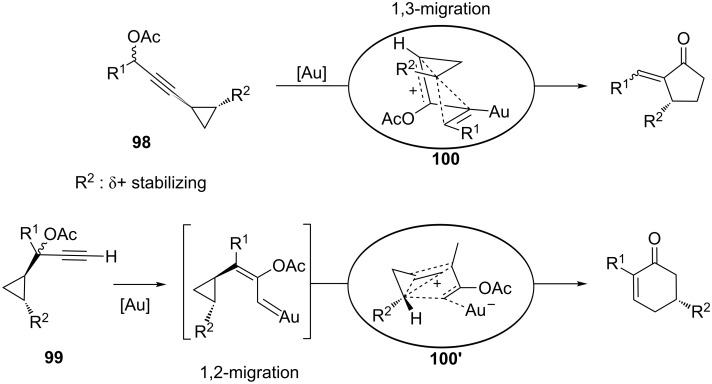
Gold-catalyzed rearrangement/cycloisomerization of cyclopropyl propargyl acetates.

In addition, 3- and 1-substituted cyclopropyl propargylic acetates **98** and **99** have also been intensively studied and provide access to 5- and 6-membered ring enones, respectively (Scheme 29) [62–64]. In the former substrates, experimental as well as computational evidence was gathered which proved the reversible nature of the [3,3]-rearrangement in these cyclopropane probes. However, these transformations proved to be stereospecific in nature through gold-stabilized non-classical carbocations **100** and **100'**, even if the stereochemical information transfer to the product is sometimes incomplete. This may arise due to a competitive gold-promoted cyclopropyl ring opening/epimerization/ring closure, both in *cis* and *trans*-cyclopropyl settings, which competes with the cyclization event, thus eroding the overall transfer of stereochemical information.

## Conclusion

From the early examples reported by Gassman and de Meijere, the field of gold-catalyzed ring expansions has experienced a continuous and sustained growth. Recently, the development of chiral gold catalysts, and the implementation of highly stereocontrolled transformations, has opened up the avenue for the application of these methodologies into more complex settings, such as natural product synthesis. In summary, gold-catalyzed ring expansions of strained rings can now be considered a mature tool for the construction of molecular complexity and thus are to be incorporated in to the toolbox of the synthetic organic chemist.
